# "MY PKU": increasing self-management in patients with phenylketonuria. A randomized controlled trial

**DOI:** 10.1186/1750-1172-6-48

**Published:** 2011-06-27

**Authors:** Amber E ten Hoedt, Carla EM Hollak, Carolien CA Boelen, N Ada P van der Herberg-van de Wetering, Nienke M ter Horst, Cora F Jonkers, Frits A Wijburg, Annet M Bosch

**Affiliations:** 1 Department of Pediatrics, Academic Medical Center (AMC), University of Amsterdam, Meibergdreef 9, 1105 AZ Amsterdam, The Netherlands; 2 Department of Internal Medicine, Academic Medical Center (AMC), University of Amsterdam, Meibergdreef 9, 1105 AZ Amsterdam, The Netherlands; 3 Department of Pediatrics, Leiden University Medical Center (LUMC), Albinusdreef 2, 2333 ZA Leiden, The Netherlands; 4 Department of Dietetics, Leiden University Medical Center (LUMC), Albinusdreef 2, 2333 ZA Leiden, The Netherlands; 5 Department of Dietetics, Academic Medical Center (AMC), University of Amsterdam, Meibergdreef 9, 1105 AZ Amsterdam, The Netherlands

## Abstract

**Background:**

Phenylketonuria (PKU) is an autosomal recessive disorder of phenylalanine metabolism. The inability to convert phenylalanine (Phe) into tyrosine causes Phe to accumulate in the body. Adherence to a protein restricted diet, resulting in reduced Phe levels, is essential to prevent cognitive decline. Frequent evaluation of plasma Phe levels and, if necessary, adjustment of the diet are the mainstay of treatment. We aimed to assess whether increased self-management of PKU patients and/or their parents is feasible and safe, by providing direct online access to blood Phe values without immediate professional guidance.

**Methods:**

Thirty-eight patients aged ≥ 1 year participated in a 10 month randomized controlled trial. Patients were randomized into a study group (1) or a control group (2). Group 2 continued the usual procedure: a phone call or e-mail by a dietician in case of a deviant Phe value. Group 1 was given a personal "My PKU" web page with a graph of their recent and previous Phe values, online general information about the dietary treatment and the Dutch PKU follow-up guidelines, and a message-box to contact their dietician if necessary. Phe values were provided on "My PKU" without advice. Outcome measures were: differences in mean Phe value, percentage of values above the recommended range and Phe sample frequency, between a 10-month pre-study period and the study period in each group, and between the groups in both periods. Furthermore we assessed satisfaction of patients and/or parents with the 'My PKU' procedure of online availability.

**Results:**

There were no significant differences in mean Phe value, percentage of values above recommended range or in frequency of blood spot sampling for Phe determination between the pre-study period and the study period in each group, nor between the 2 groups during the periods. All patients and/or parents expressed a high level of satisfaction with the new way of disease management.

**Conclusions:**

Increased self-management in PKU by providing patients and/or parents their Phe values without advice is feasible and safe and is highly appreciated.

**Trial registration:**

The trial was registered with The Netherlands National Trial Register (NTR #1171) before recruitment of patients.

## Background

Phenylketonuria (PKU; MIM 261600) is an autosomal recessive inborn error of phenylalanine metabolism caused by a deficiency of the enzyme phenylalanine hydroxylase (PAH; EC 1.14.16.1). Consequently phenylalanine (Phe) cannot be converted to tyrosine and accumulates in the body. Untreated PKU results in severely retarded mental development and neurological sequelae. These complications can effectively be prevented with a strict and unpalatable diet, severely limiting Phe intake from natural protein and supplementing all other amino acids in a mixture with vitamins and minerals [1]. Patients' blood Phe levels are frequently measured and dietary Phe intake is continuously adjusted on the basis of the results to keep Phe levels within the advised range.

The concurrent Phe level and the mean lifetime Phe level are predictive for the cognitive outcome in patients diagnosed by newborn screening [2]. In adults, elevated Phe levels are associated with executive function deficits [3]. Although there is still controversy about the safe upper limit of Phe levels during adult life, most current consensus guidelines support a diet for life [4-7]. Therefore, measurement of blood Phe levels to evaluate dietary adherence and to adjust therapy, is the cornerstone of the management of patients with PKU irrespective of age. However, maintaining Phe levels within the advised ranges is highly demanding and intensive dietary education, family involvement, promotion of self-reliance and self-efficacy are required [8]. A well-known approach to improve compliance in chronic disorders is increased self-management, which has gained widespread use in conditions such as diabetes [9], anticoagulation control [10] and hypertension [11]. Self-management appears to be safe and improves treatment-related quality of life [10,12]. Although little evidence exists regarding self-management in PKU, previous reports suggest that it might be a viable option [13] and might improve dietary compliance [14].

In recent years patients with PKU treated in the two study sites (AMC and LUMC) had a moderate degree of self-management. The patients and/or parents took blood samples at home at regular intervals and sent the blood-spots by mail to the laboratory. Results of Phe values were reported to the patient within a week and if values were above the recommended range, the dietician contacted the patient and discussed the value and potential causes. Alternatively, patients and/or parents could contact the dietician by phone or e-mail to obtain the results.

We hypothesized that self-management could be enhanced in this cohort by providing each individual patient with access to their own Phe values without interference of a professional. Such a procedure might encourage patients and/or parents to take more control of their diet or the diet of their child, in relationship to the Phe levels. Therefore, we performed an open-labeled randomized controlled trial to evaluate the safety and feasibility of online availability of individual Phe levels to PKU patients and/or their parents, on plasma Phe levels, frequency of blood sampling and satisfaction.

## Methods

### Participants

Patients were recruited via treating physicians in the two participating Dutch metabolic centers (AMC and LUMC). Patients were enrolled in the study between August 2008 and October 2008. Patients with PKU, diagnosed by newborn screening and continuously treated with a protein restricted diet and supplementation of amino acids, were eligible for inclusion in this study. Both patients with classical PKU (untreated Phe level > 1000 μmol/L, Phe tolerance < 500 mg/day) as well as with non-PKU HPA (untreated Phe level < 1000 μmol/L, Phe tolerance > 500 mg/day) could be included [15,16].

In order to ensure sufficient understanding in parents and patients of the monitoring and treatment, only patients of 1 year and up were included. Exclusion criteria were no access to internet at home and pregnancy or the wish to become pregnant in the study period.

This study was approved by the Ethical Committee of the AMC, Amsterdam. The trial was registered with The Netherlands National Trial Register (NTR #1171) before recruitment of patients.

### Procedure

After inclusion in the study, patients were randomized into a study group (group 1) and a control group (group 2). During the 10 month study period, patients in group 2 maintained their usual procedure regarding information on Phe levels and advice on necessary dietary changes: patients and/or parents sent in a bloodspot for measurement of Phe. Children and/or their parents received a call by the dietician if the value was out of the advised range. They could also obtain their Phe value by making a telephone call or by sending an e-mail to the dietician. Adult patients always received their Phe value, usually by e-mail.

**Figure 1:**
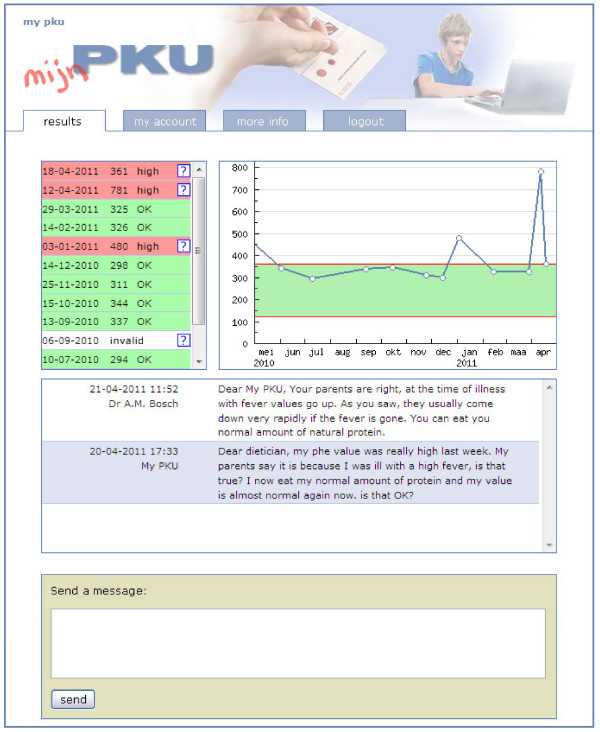
**My PKU; Phe values are in μmol/L**.

During the study period patients and/or parents in group 1 were given access to a secured personal web page on a special internet site, named 'My PKU'. In patients below 18 access information was provided to the parents. In this information letter, parents were encouraged to access the blood results information together with their children. After sending in a blood spot, patients and/or parents were alerted by an automatically generated e-mail message if a new result was available. Phe results were available at a fixed day of the week, comparable to the situation in group 2. Patients and/or parents in group 1 were not actively approached by their dietician about the Phe values if they were outside the recommended range. On their personal webpage patients and/or parents could find a graph with the most recent Phe value, clearly marked as either within or outside the recommended range, as well as an overview of the Phe values during the past 12 months (Figure 1). Also, there was a direct link to the Dutch guidelines on dietary management of PKU [17], and patients and/or parents could find information about possible causes of Phe values below or above the recommended range and on suggested actions to correct the values. In the suggested actions to correct values to the advised range, it was advised to increase or decrease phenylalanine intake, to increase caloric intake or to ensure better compliance in taking the amino acid supplements. Changes in prescribed amino acid supplements were made after consultation with the dietician only. Patients and/or parents had the opportunity to contact their dietician by entering a message in a message-box on their personal web page, which was always answered within 3 days. Importantly, patients and/or parents were assured that, same as before, they were always welcome to, and were expected to, call their treating physician or their dietician in case of a more urgent question or problem. In addition, during the study period Phe values were continuously monitored by the treating physicians and dieticians in order to be able to intervene timely in case of Phe values out of range without action taken by the patient and/or the parents.

After the study period, all patients and/or parents of group 1 continued the 'My PKU' procedure of online availability. From that time 'My PKU' was also offered to the patients and/or parents of group 2.

### Randomization

This was an open-labeled trial. Patients were randomly assigned to one of the two groups: one group with online access to the Phe values (group 1) and one control group (group 2). Randomization was performed in a 1:1 ratio by a computerized program (TENALEA Clinical Trial Data Management System) using the (non-deterministic) minimization method as described by Pocock and Simon [18]. Patient factors (strata) did not influence the allocation scheme.

### Measures

The primary outcome measures were the differences in mean Phe values and the percentage of Phe levels above the recommended range, between the 10 month pre-study period and the 10 month study period, in group 1 and 2. In addition, the differences in these primary measures between patients in group 1 and group 2 during the 10 month pre-study period and the 10 month study period were calculated. Because PKU patients are more vulnerable for high Phe levels at a younger age, we performed a subgroup analysis of group 1 for the age groups < 10 years old and 10 years and up. Per patient the mean Phe value in each period and the percentage of these values that were above the recommended range were calculated, these values were used in the different statistical analysis models.

The secondary outcome measure was the difference in frequency of blood sampling between the 10 month pre-study period and the 10 month study period, in group 1 and 2. Furthermore the difference in frequency of blood sampling between both groups in the pre-study period and the study period was calculated. In addition, in group 1 it was monitored whether, and within how many hours after receiving the e-mail that a new Phe value was available on 'My PKU', patients and/or parents logged on to 'My PKU'. Recommended ranges for plasma Phe values and recommended frequency of blood sampling per age group in the Netherlands are listed in Table 1.

**Table 1:** Recommended ranges for plasma Phe values and frequency of blood sampling in the Netherlands

Age	Plasma Phe (μmol/L)	Blood sampling
First year	120-360	weekly

1-4	120-360	every other week

4-12	120-360	monthly

12-15	120-600	monthly

> 15	120-600	monthly

### Patient satisfaction

To evaluate patient satisfaction with the procedure of online availability of Phe values, 6 months after the study period all patients and/or parents of group 1 were sent an e-mail in which they were asked to respond to the two following questions: Are you satisfied with the way of providing Phe values via a personal webpage? Which method of providing your Phe values do you prefer?

### Statistical analysis

The Statistical Package for Social Sciences (SPSS) Windows version 16.0 was used for all the analyses. To test whether there were differences between both groups for the parameters age, mean Phe value, sample frequency and the percentage Phe values above the recommended range, Mann-Whitney tests were performed. To test whether there were differences between both study periods, within each group, Wilcoxon signed-rank tests were performed.

## Results

### Participants

Sixty-eight patients, 24 adults and 44 children, were eligible and were invited by letter to participate in the study. Thirty-eight patients (11 adults) consented to participate and were randomized to either group 1 (22 patients) or group 2 (16 patients). One patient in group 1 became pregnant after randomization and was subsequently excluded from the study. All other patients completed the study. Group 1 consisted of 7 males (33.3%) and 14 females (66.7%), with a mean age of 15.0 years (SD 9.5, range 1-35 years). In group 2, 7 males (43.8%) and 9 females (56.2%) were included with a mean age of 10.9 years (SD 8.6, range 1-28 years). Both groups were comparable for age and sex. Group 1 consisted of 16 patients with classic PKU and 5 with non-PKU HPA. In group 2, 9 patients with classic PKU and 7 patients with non-PKU HPA were included. The group of patients who did not participate in the study did not differ significantly from the study group for the parameters age and sex.

### Phe values

**Figure 2:**
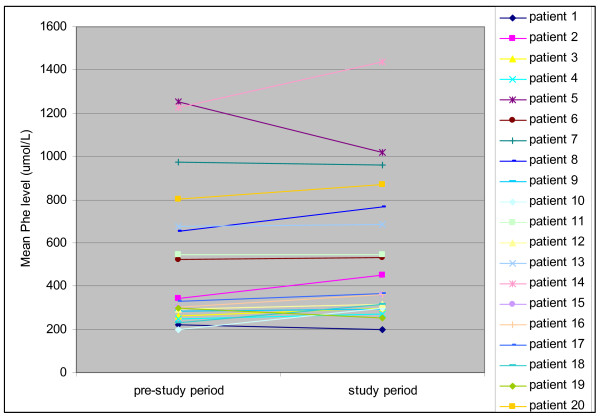
**Individual mean Phe values in the pre-study period and study period**.

The median and the range of Phe values and the percentage of Phe values above the recommended range in the pre-study period and the study period for both groups are reported in Table 2. One adult patient did not send in any bloodspot, neither in the pre-study period nor in the study period. For this reason the patient is not included in Figure 2 and was excluded from the statistical evaluation of the Phe values but was included in the analysis of the sample frequency.

**Table 2:** Median Phe values (range) and % Phe values above recommended range

	Measurement	Pre-study	Study	P-value (pre-study vs study)
**Group 1**	Median Phe value (μmol/L)	336 (198-1330)	408 (200-1438)	0.093

**Group 1**	% Phe values above rec. range	44%	49%	0.13

Group 1 < 10 years	Median Phe value (μmol/L)	273 (198-298)	295 (200-317)	0.46

	% Phe values above rec. range	15%	19%	0.35

Group 1 ≥ 10 years	Median Phe value (μmol/L)	599 (231-1330)	615 (271-1438)	0.16

	% Phe values above rec. range	57%	63%	0.16

**Group 2**	Median Phe value (μmol/L)	366 (144-1201)	381(173-1367)	0.96

**Group 2**	% Phe values above rec. range	48%	52%	0.40

Group 1 = online availability (n = 20)

Group 2 = usual procedure (n = 16)

In group 1 there was no statistically significant difference between the pre-study mean Phe value and the mean Phe value during the study period. Individual mean Phe values in both periods are depicted in Figure 2. Patients in group 2 also demonstrated no significant change in mean Phe levels between the pre-study period and the study period. No significant difference was found between the mean Phe values of group 1 and 2 in the pre study period (p = 0.78) or in the study period (p = 0.82). The percentages of Phe values above the recommended range, taking into account the ranges for age groups, between the pre-study period and the study period did not differ in either of the groups. Furthermore, the percentages of Phe values above the recommended range were comparable between both groups in the pre-study period (p = 0.83) and in the study period (p = 0.79). Group 1 subgroup analysis for the age groups < 10 years and 10 years and up showed no significant differences between the pre-study period and the study period in mean Phe value and percentage Phe values above the recommended range (Table 2).

After receiving the e-mail that a new Phe value was available on the site, patients and/or their parents logged in to their webpage within 12 hours in 163 of the total of 201 Phe values (81%). After 48 hours 172 of the 201 Phe values were checked (88%). Only 11 Phe values (5.5%) were not viewed by the patients and/or parents before a new Phe value was entered. One patient did not check 4 Phe values, 7 patients did not check one value each. In 9 out of these 11 cases, patients had an appointment with the dietician on the day the new result was available, and the dietician had provided them with their Phe value.

### Sample frequency

Results are listed in Table 3. Neither in group 1 nor in group 2 a difference in the frequency of blood sampling at home was demonstrated between the pre-study period and the study period. Because of differences in recommended frequency of sending bloodspots, the age group of 4 years and older was analyzed separately, demonstrating no significant differences between both periods. The group aged 1-4 years old consisted of only 4 patients and therefore statistical analysis was not performed on the sample frequency of this group.

**Table 3:** Median sample frequency (range) in 10 months

	Sample frequency Pre-study	Sample frequency Study	P-value (pre-study vs study)
Group 1 total	8.00 (0-29)	8.00 (0-23)	0.43

Group 1 ≥ 4 years	7.50 (0-17)	8.00 (0-23)	0.14

Group 2 total	7.50 (2-14)	8.00 (1-21)	0.36

Group 2 ≥ 4 years	6.00 (2-14)	7.00 (1-21)	0.31

Group 1 = online availability (n = 21)

Group 2 = usual procedure (n = 16)

Furthermore in both the pre-study period and the study period the mean sample frequency of both groups was comparable. Separate analysis of the age group 4 years and older showed that again group 1 and 2 were comparable in both the pre-study period (p = 0.74) as well as in the study period (p = 0.84).

### Patient satisfaction

All 22 patients and/or their parents of group 1, who were given online availability of their Phe values, responded to our questions on patient satisfaction. All reacted highly positive to the question if they were satisfied with the way of providing Phe levels via a personal webpage. The advantages of this system mentioned by the patients and/or parents were the fast and reliable availability of the results, regardless of values being within or above the recommended range, and the relatively direct contact with the dietician through the message-box. Furthermore patients and/or parents judged the overview of the Phe values and the corresponding graph positively. All patients and/or parents preferred the new way of providing Phe values.

## Discussion

This is the first randomized controlled trial evaluating the effect of increased self-management on plasma Phe levels and frequency of blood sampling in patients with PKU.

As Phe levels, lifetime and concurrent, are the most important determinants for the cognitive outcome of patients with PKU the main issue in this study was to assess the safety and feasibility of the increased self management for the patients and/or their parents. Our results clearly demonstrate that the increased self management does not negatively affect the mean Phe value, the percentage of Phe values outside the recommended range, or the sample frequency. These results partially confirm the outcome of an uncontrolled study in which a comparable way of increased self-management was investigated. In this 6 month study, patients themselves decided on frequency of blood sampling and adjustments of their diet after the Phe results were provided to them without interpretation or advice. The results demonstrated a higher mean Phe value but a stable percentage of samples above the recommended range during self management [13].

In the evaluation of group 1 as a whole, there was a no statistical difference in mean Phe levels between the study periods. However, we did find a trend (p 0.093) that Phe levels increased during online access. This may be explained by the fact that 4 patients became 12 years old during the study. At the age of 12, according to the Dutch guidelines, the recommended range is raised from 360 μmol/L to 600 μmol/L and usually the patients will increase the Phe intake and the level rises around or just before the 12^th ^birthday. This hypothesis was confirmed by the fact that in subgroup analysis of patients below 10 the trend of increasing Phe levels was not found. As very few patients below age 4 were included in the study, and because this is a vulnerable age for high Phe levels, future studies are warranted for this particular age group.

In PKU, the determinants of compliance include the frequency of taking blood samples, the mean Phe value and the percentage of Phe levels above the recommended range. The relatively high percentage of the Phe values above the recommended range during both study periods in both groups is comparable to results from other studies [13,19,20].

While compliance did not decrease during the intervention period, we did not demonstrate the increase of compliance that might be expected from increased self-management [14]. Possibly it may take longer than the 10 month study period to incorporate the new approach into the daily practice of the patients and/or their parents and to develop a full understanding of the relationship of Phe values to food intake and illness in this setting.

In other chronic disorders the quality of life (QoL) of patients has been reported to improve following self management interventions [10,21]. Hypotheses for this improvement are enhanced self-efficacy [22-24] and increased confidence and control resulting from a better education and understanding of their illness. Because no PKU specific QoL questionnaires exist and generic questionnaires have been proven not to detect abnormalities in PKU patients, QoL has not been evaluated in this study.

Parents and/or parents, without exception, preferred the new approach of providing Phe values and appreciated the direct availability of their own Phe values, the overview of the past values and the possibility to have a relatively direct contact with the dietician. Patients and/or parents interest in a more direct availability of Phe values was manifested by the high percentage of Phe values, checked within 12 hours after reporting on their "My PKU" web page. The new approach enables patients and/or their parents to make their own decisions about when to take blood samples and how to adjust the diet. Thereby it provides patients and/or parents more control on their own disease and less interference with their personal lifestyle. Remarkably, using the message-box especially adolescents provided more information on their dietary habits and asked more questions than during the outpatient visits. Possibly, this means of communication which this age group is highly accustomed to, is more comfortable for them than a personal discussion. Furthermore, the message-box is increasingly used by treating physicians to record issues and plans discussed during the outpatient visits, so that the patient and/or parents have the possibility to check it at home anytime.

## Conclusion

This randomized controlled trial is the first to demonstrate that increased self-management in PKU patients and/or their parents by providing Phe values online is a feasible, safe method and is highly appreciated.

## Competing interests

AMB is a member of a board of experts in metabolic disorders for Nutricia Liverpool. FAW is a member of the medical advisory board of Nutricia (the Netherlands) for special products.

All other authors declare that they have no conflict of interests.

## Authors' contributions

AEH, CEMH, FAW and AMB participated in the conception and design of the study. AEH, CCAB, NAPH, NMH and CFJ were involved in the acquisition of the data. AEH performed the statistical analysis and AEH, CEMH, AMB and FAW were involved in the interpretation of data. Drafting the manuscript was performed by AEH, CEMH, FAW and AMB. All authors have given final approval for the final version to be published.
